# Temporal and geographical trends in infant, neonatal and post-neonatal mortality in Italy between 1991 and 2009

**DOI:** 10.1186/1824-7288-39-19

**Published:** 2013-03-19

**Authors:** Laura Dallolio, Jacopo Lenzi, Maria Pia Fantini

**Affiliations:** 1 Department of Biomedical and Neuromotor Sciences, Alma Mater Studiorum – University of Bologna, via San Giacomo 12, Bologna, 40126, Italy

**Keywords:** Infant mortality, Italy, Disparities

## Abstract

**Background:**

Infant mortality is a key indicator of child and population health. The aim of this study is to analyse the trends in infant mortality rates (IMRs) and their components (neonatal mortality rates-NMRs and post-neonatal mortality rates-PNMRs) from 1991 to 2009 both at the national level and across the three Italian large geographical macro-areas (North, Center, South).

**Methods:**

Using data extracted from the Health for All-Italy database, IMRs, NMRs and PNMRs were calculated for the 19 Italian Regions and 2 Autonomous provinces for the years 1991–2009. Relative risks and attributable fractions were calculated for Southern and Central Italy compared with Northern Italy. Temporal trends were analysed using the robust polynomial Poisson regression models.

**Results:**

During the study period there was a 54% decline in IMR (from 7.72/1000 to 3.55/1000), a 57% decline in NMR (from 5.87/1000 to 2.55/1000) and a 46% decline in PNMR (from 1.85/1000 to 1/1000). In particular, we found a strong decline in IMRs and NMRs from 1991 to 2000/2001, and a weaker decline starting from 2002/2003. Moreover, we found a slight decrease in PNMRs until 2001/2002, and no significant variations starting from 2003. Despite these reductions, important geographical variations persisted: in 2006–2009, the most recent data available, the excess of infant mortality in Southern Italy compared with the North was 27%.

**Conclusions:**

During the period 1991–2009 Italy experienced a significant decline in IMRs, NMRs and PNMRs. We observed the same pattern for the temporal trends of these indicators across the North, the Center and South of Italy. Despite this decline, geographical disparities persisted.

## Background

The infant mortality rate (IMR), defined as the annual number of deaths in children under 1 year of age per 1000 live births, is an important indicator of child health [1]. It is also considered as a key indicator of population health, being associated with socio-economic conditions, quality of and access to medical care [2]. For this reason IMRs are interpreted as standard measures of public health and economic development and for a long time they have been used as country or regional level proxy indicators of socio-economic status [3]. Infant deaths are seen as attributable to a range of hierarchical determinants that may be proximal (e.g. maternal factors, nutrient deficiency, infections, injuries, health services utilization), intermediate (e.g. access to food, safe water, health services, vaccinations), or distal (e.g. education, employment, national income, income distribution, public health spending) [4].

Infant mortality (IM) includes neonatal (less than 28 days after birth) and post-neonatal (28 days to 11 months after birth) deaths. Neonatal mortality (NM) is more linked to biological factors such as congenital anomalies and it is particularly sensitive to proximal determinants (i.e. lifestyles of the mother during the prenatal period, mode of delivery and the care given to mothers and their babies). Post-neonatal mortality (PNM) is more influenced by distal determinants, parental circumstances (like education and income) and the care they provide for their infant [3]. From 1970 to 2010 worldwide neonatal and post-neonatal mortality rates have respectively declined by 2.1% and 2.2% per year [5]. However, IMRs and their components still remain dramatically high in several countries and the reduction by two-thirds of the infant and under-five mortality is the fourth target of the WHO Millennium Development Goals [6].

Italy also registered a remarkable reduction in infant mortality, declining from 166.8/1000 in 1901 to 7.9/1000 in 1991 [7]. Even during the period 1991–2005 IMR has continued to decrease significantly from 7.8/1000 to 3.9/1000 [8,9]. Despite this overall improvement, the reduction occurred at different rates in different geographical areas generating disparities between the Northern and Southern regions. In particular during the period 2001–2005 the excess of mortality in the South compared with the North was 37% [9].

Given the growing global and national interest in fighting health and socio-economic disparities, monitoring IMRs could represent an important first step to identify coverage gaps and successful strategies in reducing inequities.

The aim of this study is to analyse the trends in IMR and its components from 1991 to 2009 at the national level and across the three Italian large geographical macro-areas (North, Center, South). This study extends the results of a previous paper including the analysis in the years 2007, 2008 and 2009 [9].

## Methods

Data on infant, neonatal and post-neonatal deaths and on live births were retrieved from the Health for All-Italy database for the 19 Italian Regions and the 2 Autonomous Provinces of Trento and Bolzano for the study period 1991–2009 [10]. Regions were aggregated in three macro-areas (North: Piedmont, Aosta Valley, Lombardy, Trentino-South Tyrol, Veneto, Friuli-Venezia Giulia, Liguria, Emilia-Romagna; Center: Tuscany, Umbria, Marche, Lazio; South: Abruzzo, Molise, Campania, Apulia, Basilicata, Calabria, Sicily, Sardinia).

As IMRs fluctuate substantially from year to year because of the small number of infant deaths that occur per each year, in order to provide a more robust indication of the level of mortality we calculated average IMR, NMR and PNMR over 3 years.

Temporal trends were assessed using the robust polynomial Poisson regression models. We decided to use this analytical strategy in order to control for the effect of potential outliers, and added polynomial terms to the models to investigate the presence of curvilinear trends [11].

Relative risks (RR) and attributable fractions (AF %) were calculated for Central and Southern versus Northern Italy in 5-year intervals. We used these epidemiological indicators because they have been recently proposed for evaluating health disparities in high-income OECD countries [12].

## Results and discussion

Table 1 shows infant, neonatal and post-neonatal mortality rates in Italy from 1991 to 2008. During the study period there was a 54% decline in IMR (from 7.72/1000 to 3.55/1000), a 57% decline in NMR (from 5.87/1000 to 2.55/1000) and a 46% decline in PNMR (from 1.85/1000 to 1/1000). Neonatal deaths accounted for about two-thirds of all infant deaths.

**Table 1:** Infant, neonatal and post-neonatal mortality rates in Italy by 3-year intervals, years 1991–2008

	**Infant mortality**			**Neonatal mortality**			**Post-neonatal mortality**		
**Years**	**Deaths**	**Rate**	**95% CI**	**Deaths**	**Rate**	**95% CI**	**Deaths**	**Rate**	**95% CI**
1991–1993	12,965	7.72	7.58–7.85	9,862	5.87	5.75–5.98	3,103	1.85	1.78–1.91
1994–1996	10,014	6.31	6.18–6.43	7,502	4.73	4.62–4.84	2,512	1.58	1.52–1.65
1997–1999	8,502	5.37	5.26–5.49	6,346	4.01	3.91–4.11	2,156	1.36	1.31–1.42
2000–2002	7,248	4.51	4.41–4.62	5,320	3.31	3.22–3.40	1,928	1.20	1.15–1.25
2003–2005	6,410	3.91	3.82–4.01	4,647	2.84	2.76–2.92	1,763	1.08	1.03–1.13
2006–2008	5,897	3.55	3.46–3.64	4,297	2.55	2.47–2.62	1,690	1.00	0.96–1.05

CI, confidence interval.

*Source: ISTAT. Health for All-Italia. Year 2012. (*http:// http://www.istat.it/sanita/Health/ *).*

**Figure 1:**
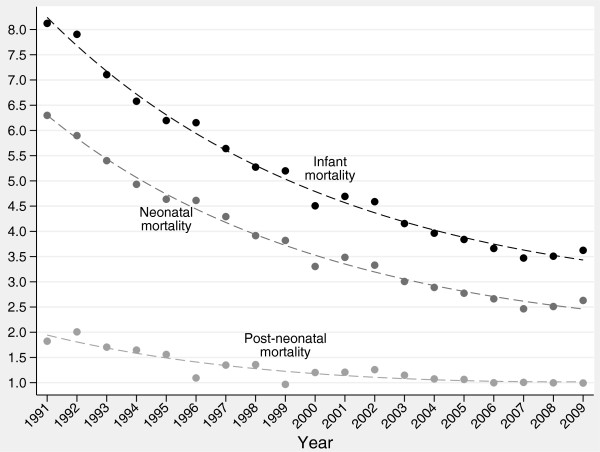
**Trends in infant, neonatal and post-neonatal mortality rates in Italy, years 1991–2009.** Dashed lines: quadratic regression slopes. Source: ISTAT. Health for All-Italia. Year 2012. (http://www.istat.it/sanita/Health/).

Polynomial regression analysis revealed the presence of a quadratic trend in IMRs, NMRs and PNMRs in Italy from 1991 to 2009 (Figure 1). In particular, we found a strong decline in IMRs and NMRs from 1991 to 2000/2001, and a weaker decline starting from 2002/2003. Moreover, we found a slight decrease in PNMRs until 2001/2002, and no significant variations starting from 2003.

This trend was similar across the three geographical macro-areas (Table 2). However important geographical variations were present: in 2006–2009 the excess of infant mortality in Southern Italy compared with the North was 27%.

**Table 2:** Infant, neonatal and post-neonatal mortality rates by 5-year intervals and geographical areas in Italy, years 1991–2009

**Geographical area**	**Infant mortality**			**Neonatal mortality**			**Post-neonatal mortality**		
	**Rate**	**RR**	**95% CI**	**Rate**	**RR**	**95% CI**	**Rate**	**RR**	**95% CI**
	**1991–1995**								
North	5.78	1		4.08	1		1.69	1	
Center	7.19	1.24	1.19–1.3	5.46	1.34	1.27–1.40	1.73	1.02	0.94–1.11
South	8.40	1.45	1.41–1.5	6.59	1.61	1.56–1.68	1.81	1.07	1.00–1.14
	**1996–2000**								
North	4.31	1		3.06	1		1.25	1	
Center	5.55	1.29	1.23–1.35	4.15	1.36	1.28–1.43	1.40	1.12	1.02–1.23
South	6.32	1.46	1.41–1.52	4.85	1.59	1.52–1.66	1.46	1.17	1.09–1.26
	**2001–2005**								
North	3.54	1		2.50	1		1.04	1	
Center	4.15	1.17	1.11–1.24	2.98	1.19	1.12–1.27	1.17	1.13	1.02–1.24
South	4.84	1.37	1.31–1.42	3.64	1.46	1.38–1.53	1.20	1.15	1.07–1.25
	**2006–2009**								
North	3.15	1		2.28	1		0.87	1	
Center	3.73	1.18	1.11–1.25	2.62	1.15	1.07–1.23	1.10	1.26	1.14–1.42
South	4.01	1.27	1.21–1.33	2.90	1.27	1.20–1.34	1.11	1.28	1.16–1.40

RR, relative risk.

*Source: ISTAT. Health for All-Italia. Year 2012.* (http:// http://www.istat.it/sanita/Health/ *).*

Analysis of attributable fractions revealed that the North–South gap was smaller in 2006–2009 compared with 1991–1995. In fact, the percentage of infant and neonatal deaths attributable to birth area (South versus North) declined respectively from 31.23% to 21.45% and from 38.05% to 21.38%. On the contrary, the percentage of post-neonatal deaths increased from 6.40% to 21.62% (Table 3).

**Table 3:** Attributable fraction (%) of infant, neonatal and post-neonatal mortality between Northern and Southern Italy

	**South – North**			
	**1991–1995**	**1996–2000**	**2001–2005**	**2006–2009**
AF – IM	31.23	31.71	26.84	21.45
AF – NM	38.05	36.94	31.30	21.38
AF – PNM	6.40	14.36	13.32	21.62

AF, attributable fraction; IM, infant mortality; NM, neonatal mortality; PNM, post-neonatal mortality.

Our findings show a sharp and constant decline of neonatal deaths for all the three macro-areas until the last years of the 1990s. This could be due to substantial improvements in the quality of care during pregnancy and in the perinatal period. In the 2000s the decrease of NM was weaker, suggesting that a sort of ceiling effect has been reached. However, the persisting differences between North and South Italy indicate that South regions should at least match the performance of North regions in the near future. Moreover, we point out that the decline of PNM was slight in the 1990s and no more present after 2003, with an alarming increase in North–South gap.

**Figure 2:**
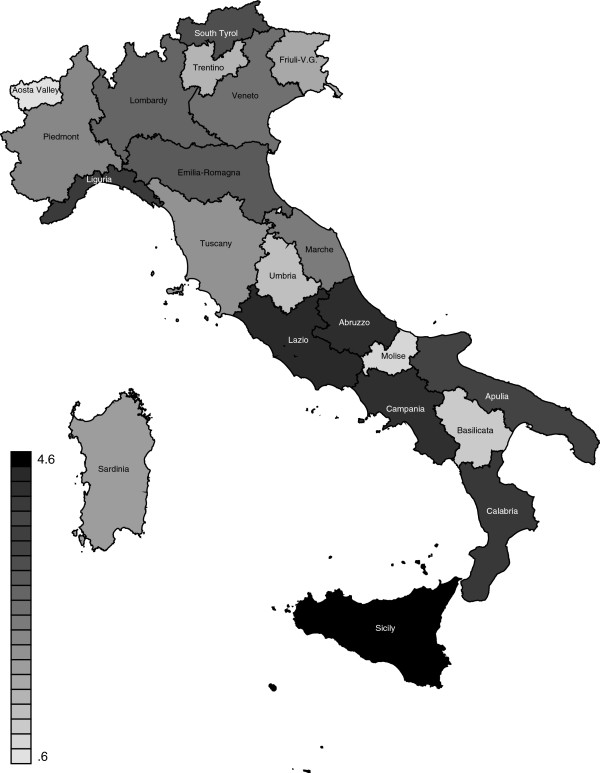
**Geographical distribution of average infant mortality rates in Italy for the years 2007–2009.** Source: ISTAT. Health for All-Italia. Year 2012. (http://www.istat.it/sanita/Health/).

Overall our data confirm low national rates of IM, NM and PNM in Italy. It is noteworthy that in 2007–2009 the IMR was one of the lowest in Europe (3.53/1000), ranging from 3.13 in Northern Italy to 3.99 in Southern Italy. As to the comparison with other European countries, we can observe that the Italian IMR is similar to that of France (3.49/1000) and Germany (3.51) in 2009. The rate in Northern Italy is lower than the national rate in Norway in 2009 (3.17), but not as low as in Sweden (2.49) and Finland (2.65). On the contrary, Southern Italy has an IMR similar to that of Netherlands (3.85) and Austria (3.79) [13]. Despite these good overall results, geographical disparities persist (Figure 2). Explanations for such disparities are complex and involve multiple factors. Determinants of infant mortality in Italy have been analysed by Parazzini and Lauria for 1980–1983 and 1989–1993 respectively [14,15]. Their findings suggested that socio-economic factors are per se important determinants of geographical disparities. More recent studies were conducted in single regions [16-18] or districts [19] but not at a national level.

A recent ecological study that examined the relationship between IM and four major socio-economic determinants found that in Italy socio-economic factors such as income, income inequality and unemployment are significantly associated with IM [20]. As Southern regions are more deprived and have a very high unemployment rate, it can be speculated that socio-economic factors do not only affect IMRs but also explain regional disparities. A North–South divide with higher rates in the more deprived North is also observed in England [21]. Analytical studies are needed to evaluate the role of distal and proximal IM determinants and their variation over time both at national and regional level. Further research could be particularly valuable in order to identify modifiable factors and to monitor regional policies and their impact on health inequalities.

## Conclusions

During the period 1991–2009 Italy experienced a significant decline in infant, neonatal and post-neonatal mortality rates. We observed the same pattern for the temporal trends of these indicators across the three geographical macro-areas (North, Center and South). Despite this decline, geographical disparities persisted.

## Competing interests

The authors declare that they have no competing interests.

## Authors’ contribution

Paper conception and drafting of manuscript: LD. Analysis and interpretation of data: JL. Critical revision: MPF. All authors read and approved the final manuscript.
